# Local buckling behavior of buried pipeline under seismic oblique-reverse fault displacement

**DOI:** 10.1038/s41598-022-24728-y

**Published:** 2022-11-22

**Authors:** Lingyue Xu, Xudong Cheng, Runkang Huang, Wendi Chen, Wenjun Hu

**Affiliations:** 1 College of Pipeline and Civil Engineering, China University of Petroleum(East China), Qingdao, 266580 China; 2 China Petroleum Pipeline Engineering Corporation, Langfang, 065000 Hebei China; 3 Institute of Mountain Hazards and Environment, Chinese Academy of Sciences, Chengdu, 610041 China

**Keywords:** Civil engineering, Tectonics

## Abstract

Seismic fault displacement is the main factor leading to local buckling failure of the buried pipeline, especially crossing the oblique-reverse fault. The local buckling behavior of the buried pipeline is complex under the 3-D displacement of the oblique-reverse fault. In this work, the pipe local buckling mechanism was discussed, then a shell and solid element nonlinear contact coupling model of the pipeline crossing oblique-reverse fault was established based on the ABAQUS program. The local buckling behavior (potential local buckling locations, developing process) of the pipeline under oblique-reverse fault displacement was systematically analyzed, comparing against the same under single fault displacement. Subsequently, the influence of internal pressure, diameter thickness ratio and burial depth on the local buckling behavior of the pipeline were discussed. The numerical results revealed two potential locations and three stages of the local buckling, then the potential local buckling locations and three stages of the local buckling under different internal pressure, diameter thickness ratio and burial depth were obtained. It proves that the local buckling of the pipeline is more sensitive to the oblique-reverse fault displacement than single fault displacement and provides a reference for the aseismic design and reinforcement of the pipeline crossing the oblique-reverse fault.

## Introduction

With the continuous development of global economy and society, the buried pipeline is widely used for the long-distance and stable transportation of oil and gas resources. Buried pipeline usually covers a wide range of regions, and the vicinity along the pipeline will inevitably suffer from various geological hazards, such as fault displacement induced by earthquakes. It poses a great threat to the safe operation of buried pipelines, especially for the oblique-reverse fault (composed fault)^1–3^. Line D of Central Asia Natural Gas Pipeline (Line D) is the typical project where the pipeline crosses the oblique-reverse fault^4^. The pipeline may be mainly bent and compressed under oblique-reverse fault displacement, resulting in local buckling failure. It should be noted that the local buckling threatens pipelines safety greatly, which can lead to the leakage of contents and weaken the stability of the pipeline structure^5–8^.

The systematic research on seismic resistance of buried pipelines started from the massive damage event caused by the San Fernando earthquake in 1971^9^. Subsequently, researchers successively carried out research on the mechanical response of buried pipelines under seismic fault displacement. Rofooei et al.^10^ conducted a full-scale test on the stress and strain of pipelines under the reverse fault displacement to determine the deformation behavior characteristics of the pipeline with a diameter of *D* = 110 mm. Demirci et al.^11^ examined the behavior of buried pipelines crossing strike-slip faults using experimental and numerical modeling and discussed the influence of pipe parameters on the pipe behavior. Kaya et al.^12^ proposed a 3-D nonlinear continuum finite element model based on the seismic response of a 2200 mm-diameter welded steel pipe at the strike-slip Kullar fault. They predicted the locations of the local buckling due to the strike-slip fault rupture. Vazouras et al.^13^ studied the response of the buried pipeline under strike-slip fault displacement and obtained critical fault displacements of pipeline failure under different operating conditions. Based on the nonlinear finite element model, Liu et al.^14^ analyzed the pipe strain response under the combination of compression and bending load when the X80 pipeline crossed the strike-slip fault and proposed a semi-empirical model for peak strain prediction. Melissianos et al.^15^ discussed the local buckling and tension cracking behavior of continuous pipeline and elbow pipelines under strike-slip fault. They proposed a three-step method for performance evaluation of buried pipelines across the fault. Gawande et al.^8^ proposed the FE model considering the interaction of the pipeline with surrounding soil by taking into account the contact between the pipeline and the soil, the nonlinear response of soil, and geometrical nonlinearities. They predicted a systematic pre-buckling pattern of a pipe undergoing strike-slip fault. Zhang et al.^7^ proposed a numerical simulation model to study the buckling evolution mechanism of the buried steel pipe under strike-skip fault and reverse fault movements. They preliminarily gave three stages of the local buckling development based on the pipeline deformation shape: elastic stage, plastic stage, and local buckling stage. Rofooei et al.^16^ made a detailed parameter analysis of the local buckling behavior under the reverse fault through test and numerical simulation, including pipeline material, diameter thickness ratio, burial depth, and diameter ratio. Yang et al.^17^ investigated the buckling failure of buried subsea pipeline under reverse fault displacement. The results show that subsea pipelines can obtain a large deformation capacity and the flexure curve shape is usually S-shape, and seven different modes of collapse and propagation are discovered in the empty waiting state. Melissianos et al.^5^ carried out an extensive parametric study on the pipeline crossing reverse fault and offered the first comprehensive attempt to quantify the qualitative criterion that deeply buried pipes with high *D*/*t* ratio tend to buckle locally, while shallowly buried pipes with low *D*/*t* ratio tend to buckle globally. Based on the shell element-nonlinear contact coupling model and design criteria, Cheng et al.^2,18^ gave the prediction formula of the maximum tensile and compressive strain of buried X80 steel pipe under the oblique-reverse fault displacement and proposed the FE model of X80 steel pipe crossing oblique-reverse fault. Then, the strain evolution of the pipeline under three failure modes (local buckling, tensile rupture, and section ovalization) was obtained, and the failure mode sequence was discussed.

Although some work has been done on the local buckling behavior of buried pipelines under the single fault displacement (reverse fault, strike-slip fault in this paper), the pipe local buckling behavior under the composed fault (oblique-reverse fault in this paper) is still unclear due to the 3-D oblique-reverse fault displacement. Compared with the single fault displacement, the pipeline is subjected the soil resistance in axial, lateral, and vertical directions during the oblique-reverse fault displacement, the potential local buckling locations of the pipeline may change accordingly. Besides, the local buckling of the pipeline as a cylindrical shell will experience an obvious stage feature under axial compression^7,19,20^. The pipe local buckling behavior (potential local buckling locations and the developing process) under single fault displacement may not accurately evaluate that under the oblique-reverse fault.

Therefore, it is significant to study the pipe local buckling behavior (including potential local buckling locations and the developing process) under oblique-reverse fault displacement, which can provide the reference for the aseismic design and the reinforcement of the pipe local buckling segment.

In view of the above analysis, the pipe local buckling mechanism is discussed, and a nonlinear numerical model of buried pipeline across the oblique-reverse fault is proposed based on parameters of Line D. Then, local buckling behavior of the buried pipeline under oblique-reverse fault displacement was studied comparing with the single fault. The potential local buckling locations were given, and three stages of the local buckling development were defined based on the local buckling mechanism and the numerical results. Subsequently, the influence of internal pressure *P*, thickness ratio *D*/*t*, and burial depth *h* on the local buckling behavior was quantitatively analyzed to determine the safer operating condition. It proves that the pipe local buckling behavior under the oblique-reverse fault is more dangerous than the single fault. The reference for the aseismic design and reinforcement of the pipeline under the oblique-reverse fault displacement in practical engineering is provided.

## Fundamental theory

### Oblique-reverse fault displacement

**Figure 1:**
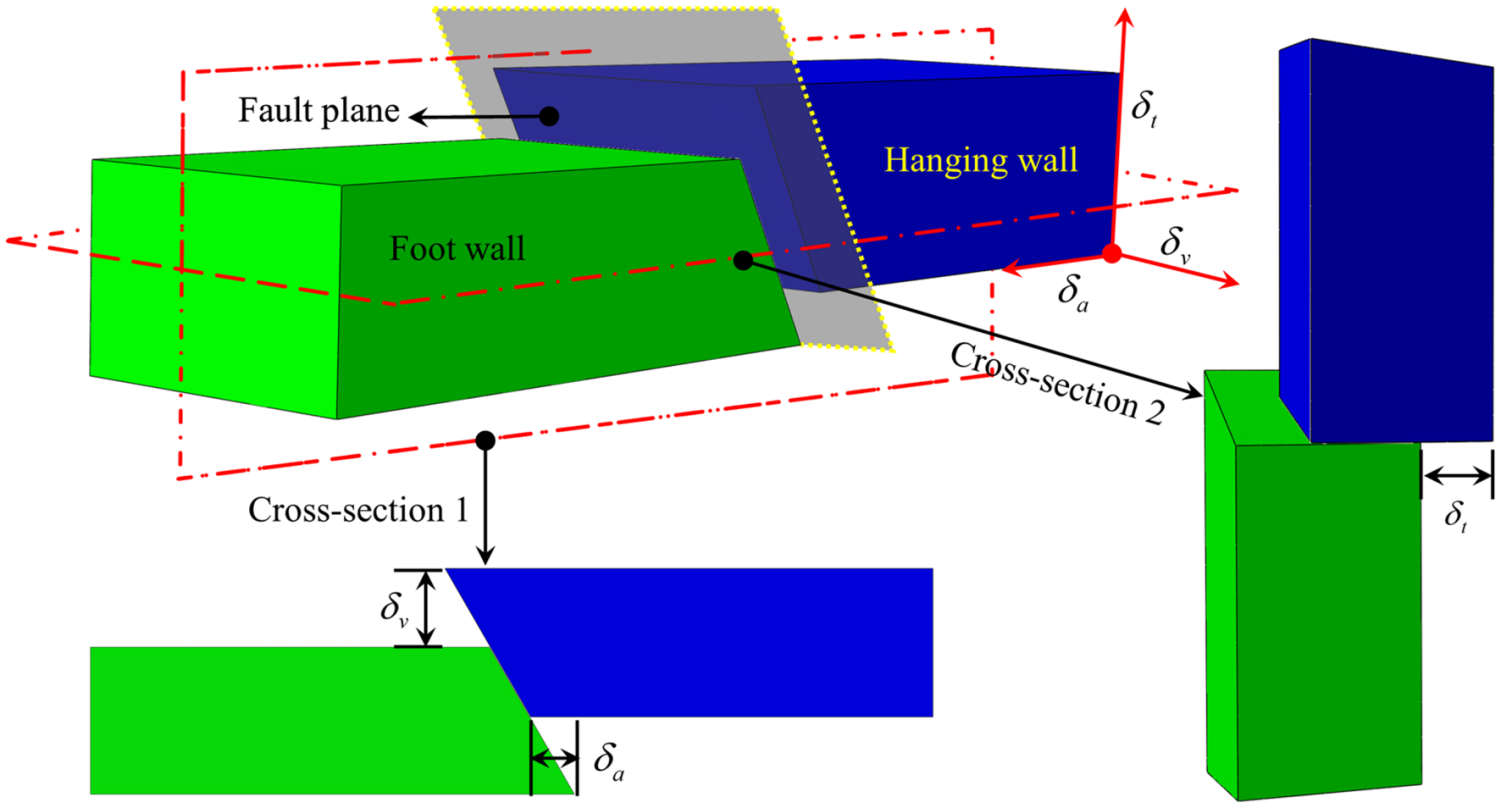
Schematic of the oblique-reverse fault displacement.

The oblique-reverse fault is the combination of the reverse fault and strike-slip fault. When the oblique-reverse fault displacement occurs, the hanging wall moves in axial, lateral, and vertical directions. According to the engineering geological reports of the Line D^4^ and Refs^21,22^, the dip-slip displacement and the strike-slip displacement of the oblique-reverse fault could be assumed as $\delta_{1} = \sqrt {\delta^{2} - \delta_{2}^{2} }$ and $\delta_{2}$ = $\delta$/2, respectively. The oblique-reverse fault displacement $\delta$ can be determined using the empirical statistical Eqs. (1) and (2) of engineering design in China and the relationship between earthquake intensity and magnitude^23–25^. The lateral displacement component *δ*_*v*_, axial displacement component *δ*_*a*_, and vertical displacement component *δ*_*t*_ of the oblique-reverse fault can be obtained from Eqs. (3)–(5), respectively. The schematic illustration of the oblique-reverse fault displacement component is shown in Fig. 1.

1 $$\lg \delta = - 3.019 + 0.4646M$$

2 $$I = 0.24 + 1.29M$$

3 $$\delta_{v} = \delta_{1} \sin \psi$$

4 $$\delta_{a} = \delta_{1} \cos \psi \sin \beta + \delta_{2} \cos \beta$$

5 $$\delta_{t} = \delta_{2} \sin \beta - \delta_{1} \cos \psi \cos \beta$$

where *M* is the earthquake magnitude; *I* is the earthquake intensity; *β* is the pipe-fault crossing angle, °; *ψ* is the fault dip angle, °. Most pipelines in Line D are in the high seismic intensity area of VIII–X, from which the range of $\delta$ can be determined as 0.59–3.13 m.

### Pipe local buckling mechanism

Pipe local buckling failure, also known as “shell mode” buckling, is a failure mode related to instability caused by compression loads or bending moments^1^. It is mainly manifested by local fold compression of the pipeline in the form of bulge or collapse, which may weaken the stability of the pipeline structure and threaten pipelines safety operation. Many formulas explain the compressive stress and the bending moment of the cylindrical shell under compression and bending. The buckling moment *M*_c_ and buckling stress *σ*_c_ corresponding to the cylindrical shell can be obtained by Eqs. (6) and (7)^26^:

6 $$M_{{\text{c}}} = \frac{{\sqrt 2 E\pi Rt^{2} }}{{\sqrt {3\left( {1 - \nu^{2} } \right)} }}$$

7 $$\sigma_{{\text{c}}} = \frac{\sqrt 2 E}{{\sqrt {3\left( {1 - \nu^{2} } \right)} }}\frac{t}{R}$$

where *R* is the radius, m; *t* is the wall thickness, m; *E* is the elastic modulus, MPa; *ν* is the Poisson ratio.

However, the bending and compression deformation of the buried pipeline subjected to the oblique-reverse fault displacement is a higher-order nonlinear problem. The pipe-soil interaction affects the buckling behavior of the pipeline. In addition, when the pipe cross-section has large deformation, the superposition principle can't be used for the interaction of axial strain and bending strain, and there may be residual stress and stress concentration in the pipeline. It is difficult to solve the pipe local buckling behavior under the oblique-reverse fault displacement by the analytical method. The experimental and numerical methods are appropriate.

Based on the experiment and FE model proposed by Peng et al.^19,20^, the local buckling failure of the cylindrical shell occurs when the cylindrical shell is subjected to a large axial compression load.

**Figure 2:**
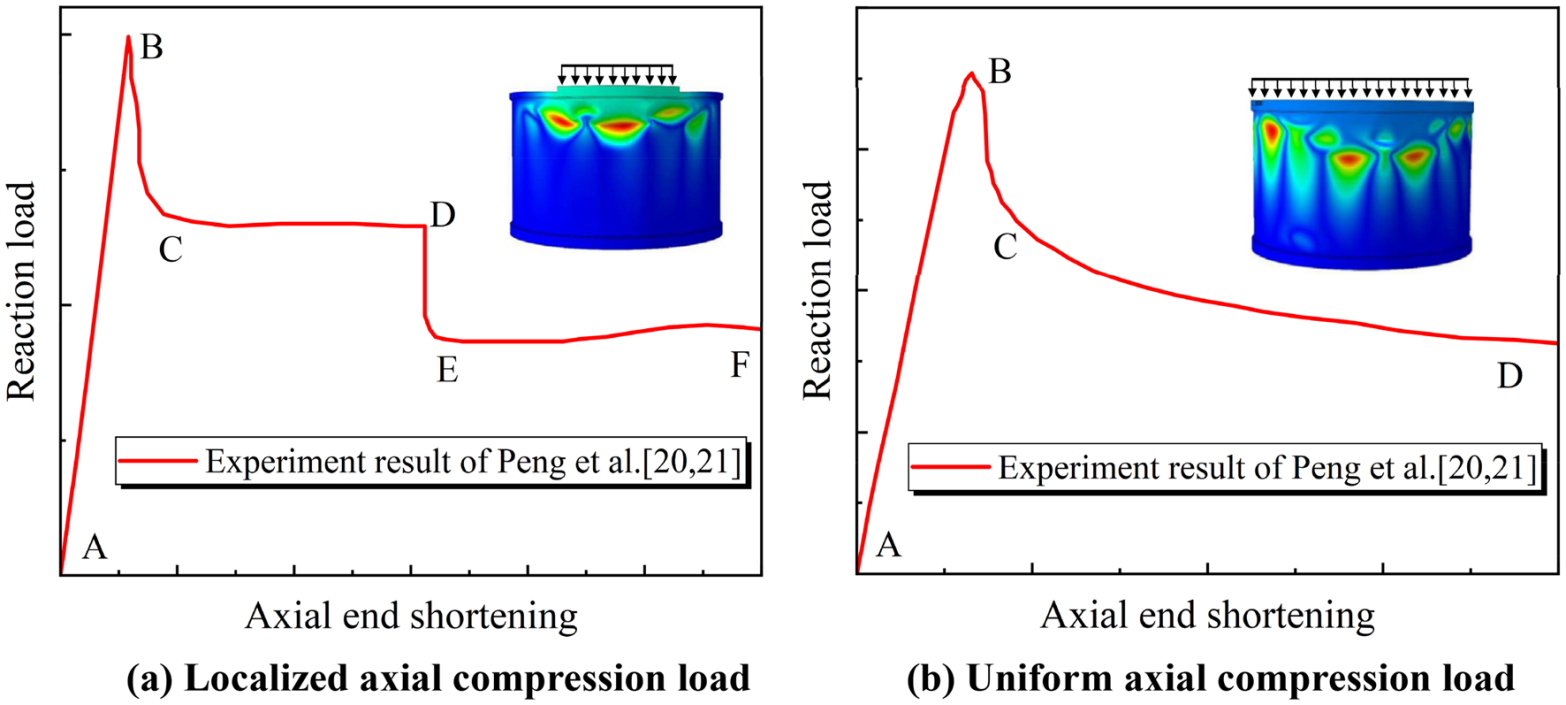
Load–displacement curve of cylindrical shell^19,20^.

According to the load–displacement curve of the cylindrical shell, when the cylindrical shell is subjected to a small distribution scope of localized axial compression load, the buckling behavior can be divided into five stages: linear elastic stage (AB), load dropped stage (BC), flow deformation stage (CD), second buckling stage (DE) and flow deformation stage (EF), as shown in Fig. 2a. When the cylindrical shell with a large distribution scope of localized axial compression load or uniform axial compression load, the buckling behavior can be divided into three stages: linear elastic stage (AB), load dropped stage (BC), flow deformation stage (CD), as shown in Fig. 2b.

It proves that the local buckling development of the cylindrical shell shows an obvious stage feature under axial compression. When the pipeline is subjected to the oblique-reverse fault displacement, the bending deformation of the pipeline will cause a part of axial compression, and the axial soil friction will cause another part of axial compression. It can be considered that the pipeline is subjected to localized axial compression load with a large distribution scope. Therefore, the local buckling of the pipeline as a cylindrical shell structure will experience similar development stages, as shown in Fig. 2b. Additionally, the bending moment under the fault displacement and the pipe internal pressure affect the local buckling development stage of the pipeline. Zhang et al.^7^ proposed a FE model of pipeline crossing the strike-slip fault and preliminarily defined three stages of the local buckling development based on the pipe deformation shape under the strike-slip fault displacement, namely elastic stage, plastic stage, and local buckling stage. It is proved that the local buckling of buried pipelines also has a stage feature^7,19,20^.

To clearly obtain the pipe local buckling development stage through the numerical method, the failure criterion for pipe local buckling should be determined.

### Failure criterion for pipe local buckling

The limit state of pipe local buckling failure depends on many factors, including pipeline type, defects, diameter-thickness ratio, internal pressure, etc. Based on the local buckling failure of pipelines caused by seismic fault displacement, relevant standards such as Canadian Standard Association (CSA-Z662-2019) and American Lifelines Alliance (ALA)^27,28^ provide the upper limit of compressive strain. When the compressive strain of the pipeline exceeds a certain limit and structural instability such as wrinkle or collapse occurs on the pipe wall, it can be regarded as the compression limit state. According to the American Lifelines Alliance (ALA)^28^, the local buckling failure criteria under two different compression limit states were given as follows:

Operable limit (onset of buckling), corresponding to 10% failure probability:

8 $$\varepsilon_{{\text{c}}}^{{{\text{crit}}}} = 0.5\frac{t}{D} - 0.0025 + 3000\left( {\frac{{\sigma_{h} }}{E}} \right)^{2}$$

9 $$\sigma_{h} = \left\{ {\begin{array}{*{20}c} {\frac{PD}{{2t}}} & {\frac{PD}{{2t\sigma_{s} }} < 0.4} \\ {0.4\sigma_{s} } & {\frac{PD}{{2t\sigma_{s} }} \ge 0.4} \\ \end{array} } \right.$$

Pressure integrity limit (pipe wall rupture), corresponding to 90% failure probability:

10 $$\varepsilon_{{\text{c}}}^{{{\text{crit}}}} = 1.76\frac{t}{D}$$

where *ε*_c_^crit^ is the critical compressive strain of the pipeline, MPa; *t* is pipe wall thickness, mm; *D* is the diameter of the pipeline, m; *σ*_h_ is the circumferential stress of the pipeline, MPa; *P* is the internal pressure of the pipeline, MPa.

For the performance goal of post-event operability, most literature suggests using the onset of buckling as the appropriate limit state. So the operable limit state corresponding to 10% failure probability was adopted as the criterion for local buckling failure judgment of buried pipeline in this study.

## Numerical modeling

### Geometric model

**Figure 3:**
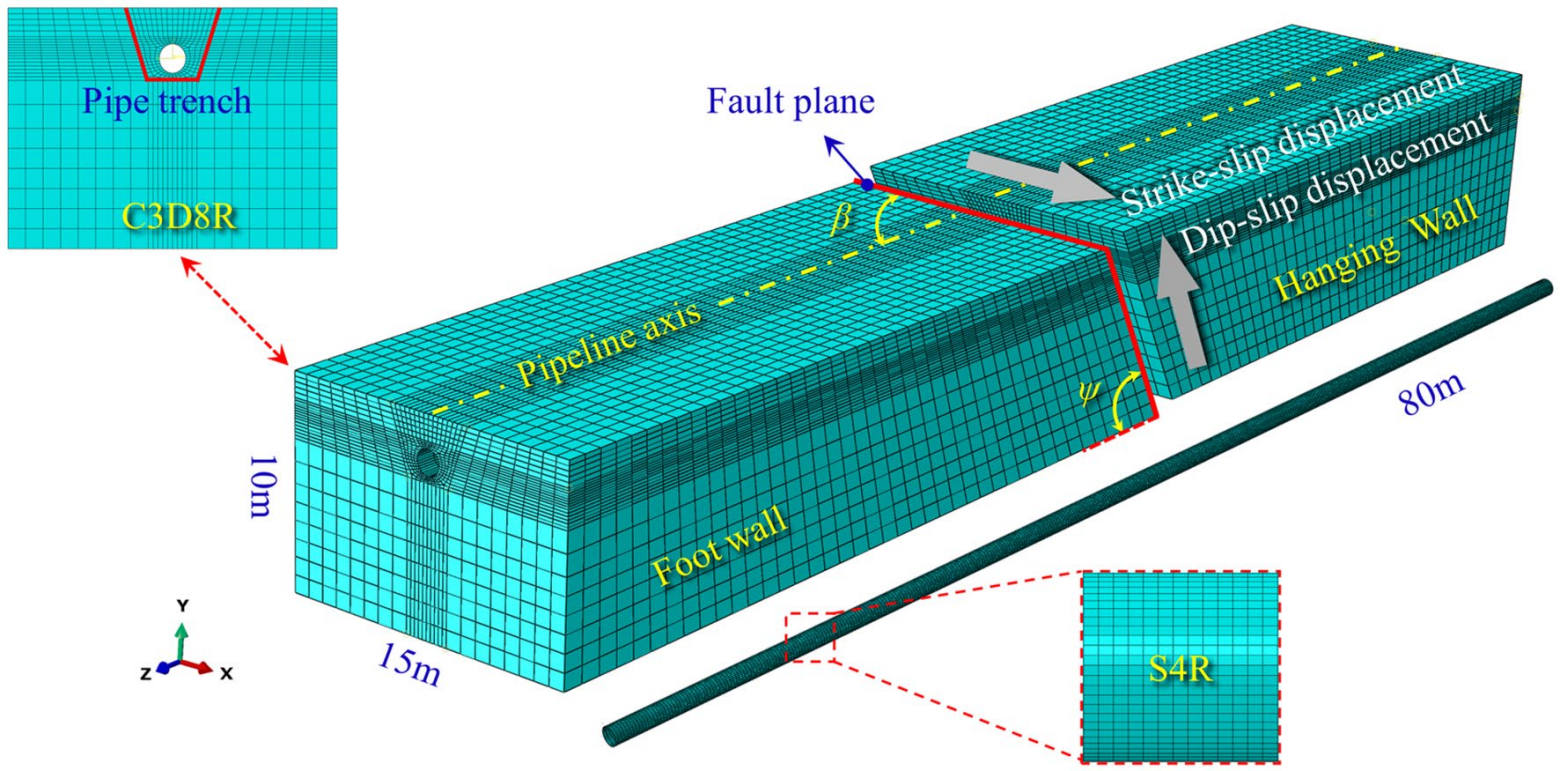
Finite element model and mesh detail.

This paper focused on analyzing the pipeline-soil interaction model with large deformation and torsional response near the fault. Based on the research of Vazouras et al.^29^, it can meet the accuracy requirements of the pipeline strain when the model dimensions in X, Y, Z directions are equal to or more than 11D, 5D, and 65D, respectively. In this paper, the dimension in Y direction is lengthened to 8D (10 m) to consider the underlying rock layer. Therefore, the model dimensions in this paper are determined as 11D, 8D, and 65D in X, Y, Z directions, respectively, that is, *H* = 10 m, *B* = 15 m, and *L* = 80 m. The FE model is shown in Fig. 3.

**Figure 4:**
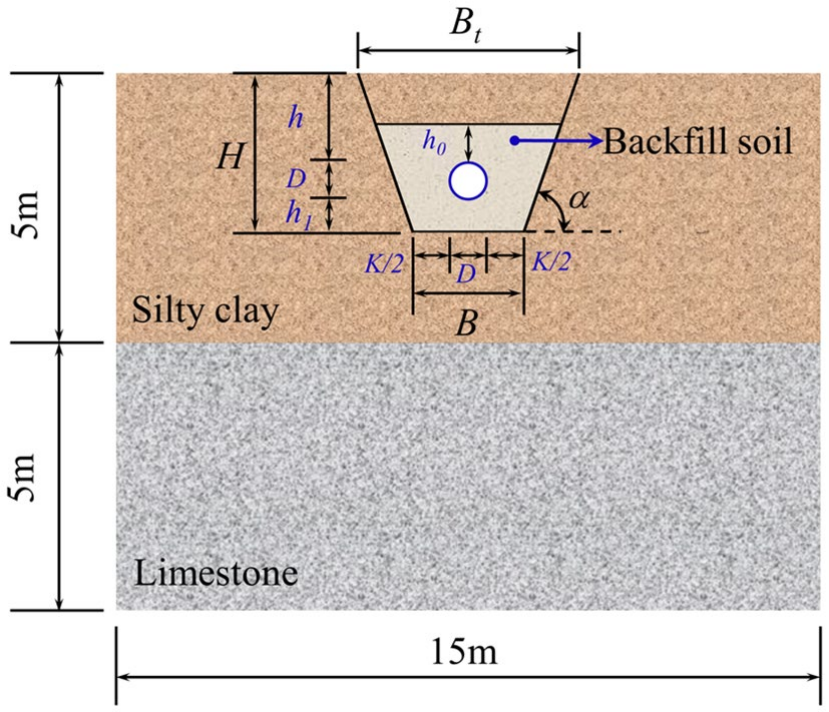
Cross-section schematic diagram of the FE model.

The pipe trench and backfill material were considered in the FE model. When the native soil is silty clay, and there is no load on the slope top, the maximum trench slope ratio is tan*α* = 3.03 based on the Design of Gas Transmission Pipeline Engineering GB50251-2015 (Chinese Standard)^30^. The thicknesses of the backfill soil above the pipeline *h*_0_ and under the pipeline *h*_1_ are no less than 0.3 m. The remaining trench space could be backfilled with native soil, silty clay in this paper. Meanwhile, the trench bottom needs to be widened and excavated to ensure the welding and laying working face, whose size can be calculated by Eqs. (11) and (12). The detailed cross-sectional size of the FE model and trench is shown in Fig. 4.

11 $$B = D + K$$

12 $$B_{t} = B + H \cdot \cot \alpha$$

where *B* is the trench bottom width, m; *B*_t_ is the trench top width, m; *D* is the diameter of the pipeline, m; *K* is the allowance for trench bottom widening (convenient for construction), m; When the trench depth *H* < 3 m, *K* = 0.8 m; When the trench depth 3 < *H* < 5 m, *K* = 1.0 m^30^.

The loads and boundary conditions of the FE model should be set appropriately to simulate the deformation of the pipeline and soil accurately.

A total of three sets of loads were applied to the FE model, and the implicit analysis was adopted. Load (1): The gravity load was applied to the entire FE model; Load (2): The operating internal pressure was applied to the inner pipeline surface to consider the initial stress and strain state of the pipe-soil system; Load (3): The hanging wall simultaneously moved along the fault plane in the axial, lateral, and vertical directions to simulate the oblique-reverse fault movement. For loads (1) and (2), the Static-General is adopted as the solving algorithm. For load (3), the Implicit-Dynamic is adopted as the solving algorithm.

The boundary conditions at the surrounding and bottom surfaces of the foot wall were fixed in X, Y, Z directions, whereas the ground surface was set free. The displacements in X, Y, Z directions were applied to the soil in the hanging wall. To simplify the numerical simulation process, applying the axial constraint at the end of the pipeline by assuming the axial strain distribution beyond the FE model was zero^14,16^. The pipe end on the foot wall was constrained in the axial direction, while the other end of the pipeline was unconstrained and could move with the hanging wall^3^.

### Material and interaction

The pipe material adopted in Line D was X80, and the X80 steel pipeline characterized by diameter *D* = 1.219 m, pipe wall thickness *t* = 22 mm, burial depth *h* = 1.2 m, and internal pressure *P* = 12 MPa was selected as the research object. Based on the local buckling failure criterion, the critical compressive strain *ε*_c_^crit^ can be determined as 1%. The Ramberg–Osgood model, whose stress–strain relationship can better meet actual pipe material, was adopted for the pipe constitutive, and the constitutive expression is shown as Eq. (13).

13 $$\varepsilon = \frac{{\sigma_{s} }}{E}\left[ {\frac{\sigma }{{\sigma_{s} }} + \alpha \left( {\frac{\sigma }{{\sigma_{s} }}} \right)^{N} } \right]$$

where *ε* is the strain of the pipeline; *σ*_*s*_ is the yield stress of the pipeline, MPa; *E* is the elastic modulus, MPa; *σ* is the stress of the pipeline, MPa; *α*, *N* are parameters of Ramberg–Osgood model. For X80 steel pipeline used in Line D, *E* = 207GPa, *σ*_*s*_ = 530 MPa, *α* = 0.86, *N* = 28^22^.

The upper stratum within 5 m under the ground was designated as silty clay, while the stratum of 5–10 m was designated as limestone, and the trench was backfilled with fine sand, as shown in Fig. 4. The specific parameters of strata are shown in Table 1. Based on the simulation experience of Refs^2,3,13–15^, the Mohr–Coulomb model is adopted for the constitutive of the silty clay, fine sand, and limestone.

**Table 1:** Physical parameters of soil strata.

Soil strata	Density	Elastic modulus	Poisson’s ratio	Cohesion	Friction angle	Dilation angle
	*ρ* (kg/m^3^)	*E* (MPa)	*ν*	*c* (kPa)	*φ* (°)	*Ψ* (°)
Silty clay	1900	33	0.27	35	22	0
Fine sand	2000	30	0.3	10	35	10
Limestone	2090	28,500	0.29	6720	42	0

**Figure 5:**
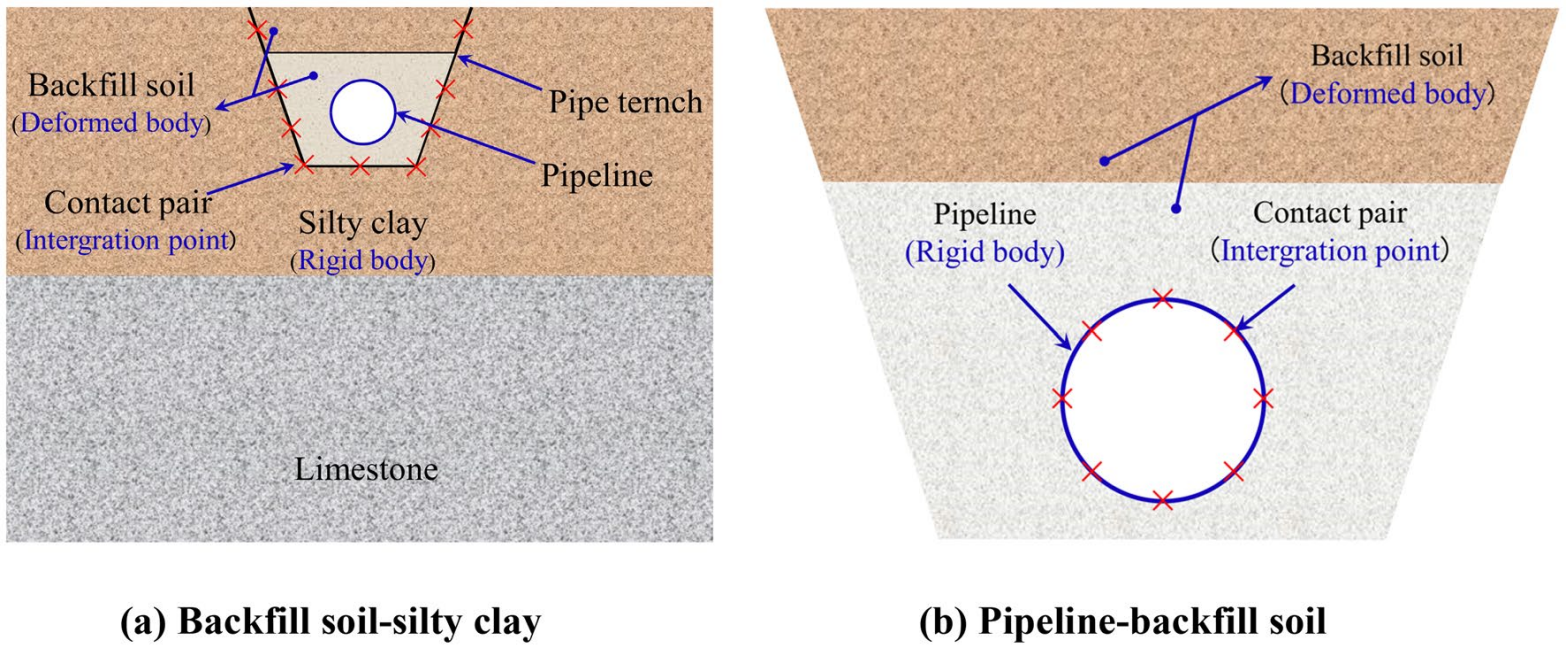
Schematic diagram of the interaction contact model.

There are two main friction interfaces in the developed FE model: backfill soil-silty clay and pipeline-backfill soil. Considering the material nonlinearity of pipeline and the state nonlinearity of soil and limestone caused by large geometric deformation. The master–slave contact algorithm in ABAQUS was used to achieve the nonlinear contact of backfill soil-silty clay (Fig. 5a) and backfill soil-pipeline (Fig. 5b)^2,3,6,7^. The penalty element was used to transfer the shear stress, and hard contact was used for normal action. The friction coefficient of backfill soil (fine sand)-silty clay and pipeline-backfill soil (fine sand) was 0.4 and 0.6, respectively^31,32^. The schematic diagram of the interaction models is shown in Fig. 5.

### Mesh densities and independence verification

S4R was selected as the mesh element for the pipeline, while C3D8R was adopted for the mesh element of soil strata in ABAQUS. To accurately capture the local buckling shape, the S4R element size along the pipeline length in the wrinkle-buckling region should be smaller than the buckling wavelength ($\lambda_{P} < \lambda_{i} = 3.44\sqrt {Dt/2} = 0.4\,{\text{m}}$)^16,33,34^. Additionally, to eliminate the influence of mesh size on local buckling behavior, 200, 400, 600, 800, 1000 mesh elements along the pipe length, 20, 40, 60, 80, 100 mesh elements along the pipe circumference were taken to study the mesh convergence.

**Figure 6:**
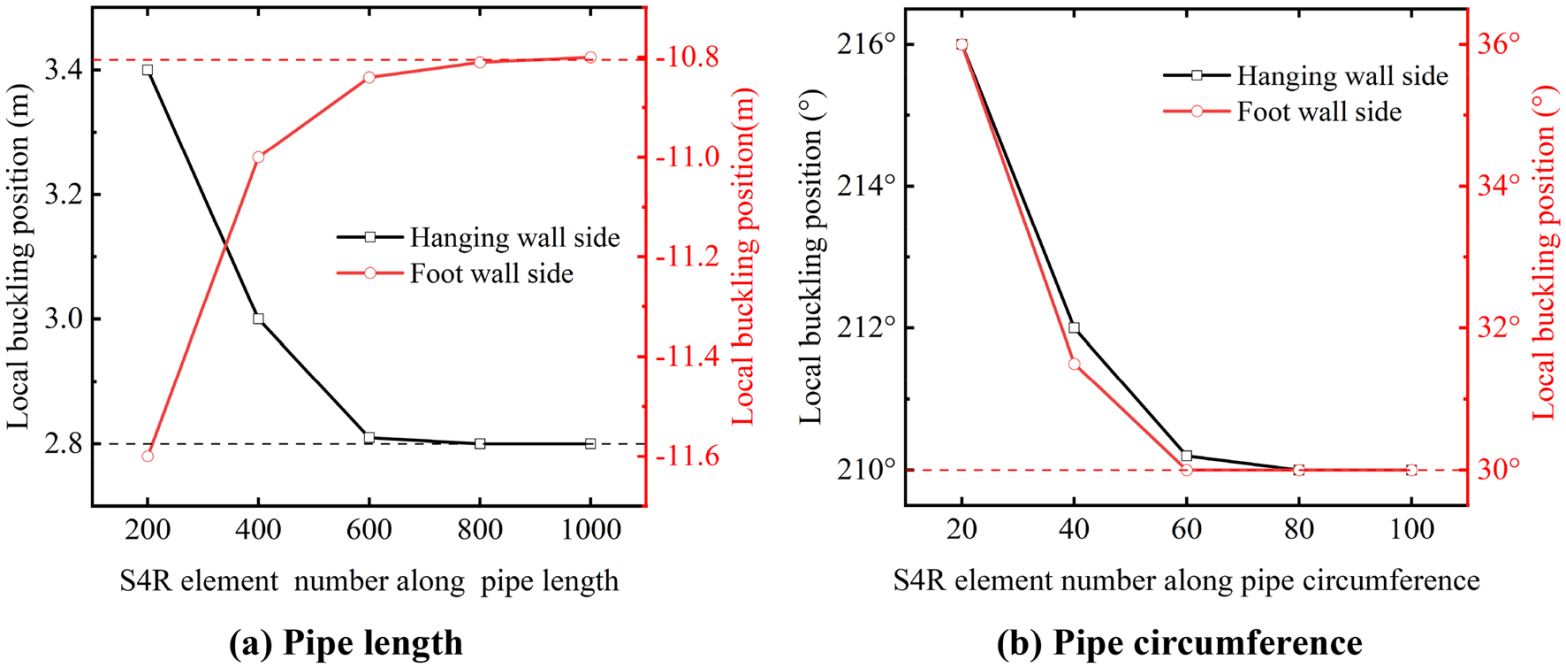
Influence of mesh density on pipe local buckling position.

As seen in Fig. 6, the local buckling positions in the hanging wall and foot wall side are convergent when the pipe length and pipe circumference elements of the pipeline exceed 800 and 60, respectively. Therefore, considering the accuracy and calculation cost of the FE model, the mesh density of the pipeline was divided into 800 parts along the pipe length and 60 parts along the pipe circumference. It should be noted that since the soil strata are not the main object of this research work, the specific details about the mesh convergence study of soil and rock will not be given here for the sake of brevity. The meshing details are shown in Fig. 3.

### Comparison and validation of numerical simulation results

Considering the difficult operability of 3-D oblique-reverse fault movement process, the immaturity of current technology and the limitations of test equipment, the experimental data, numerical simulation and conclusions related to the failure behaviors of the buried pipeline crossing oblique-reverse fault is relatively few. Therefore, the data obtained from the results of the experiment and FE simulation by Jalali et al.^35^ was used to verify the proposed FE model in this paper. The values of the relevant parameters are shown in Table 2.

**Table 2:** Parameters of the experiment by Jalali et al.^35^.

Parameter type		Parameter value	Parameter type		Parameter value
Fault	Fault dip angle, *ψ* (°)	61	Pipeline	Pipe material	API-5L Grade B
	Fault type	Reverse fault		Elastic modulus, *E* (MPa)	200
	Crossing angle,* β* (°)	90		Poisson's ratio, ν	0.3
	Displacement, $\delta$ (m)	0.6		Yield stress, *σ*_*s*_ (MPa)	241
Soil	Density, *ρ* (kg/m^3^)	1870		Length, *L* (m)	8.5
	Model size, (m × m × m)	8.5 × 1.7 × 2		Diameter, *D* (mm)	114.3
	Friction angle, *φ* (°)	33.5		Thickness, *t* (mm)	4.4
	Dilation angle, *Ψ* (°)	3.5		Diameter thickness ratio, *D*/*t*	38
	Elastic modulus, *E* (MPa)	33		Internal pressure, *P* (MPa)	0
	Poisson's ratio, ν	0.3		Burial depth,* H* (m)	1
	Cohesion, *c* (kPa)	5			

**Figure 7:**
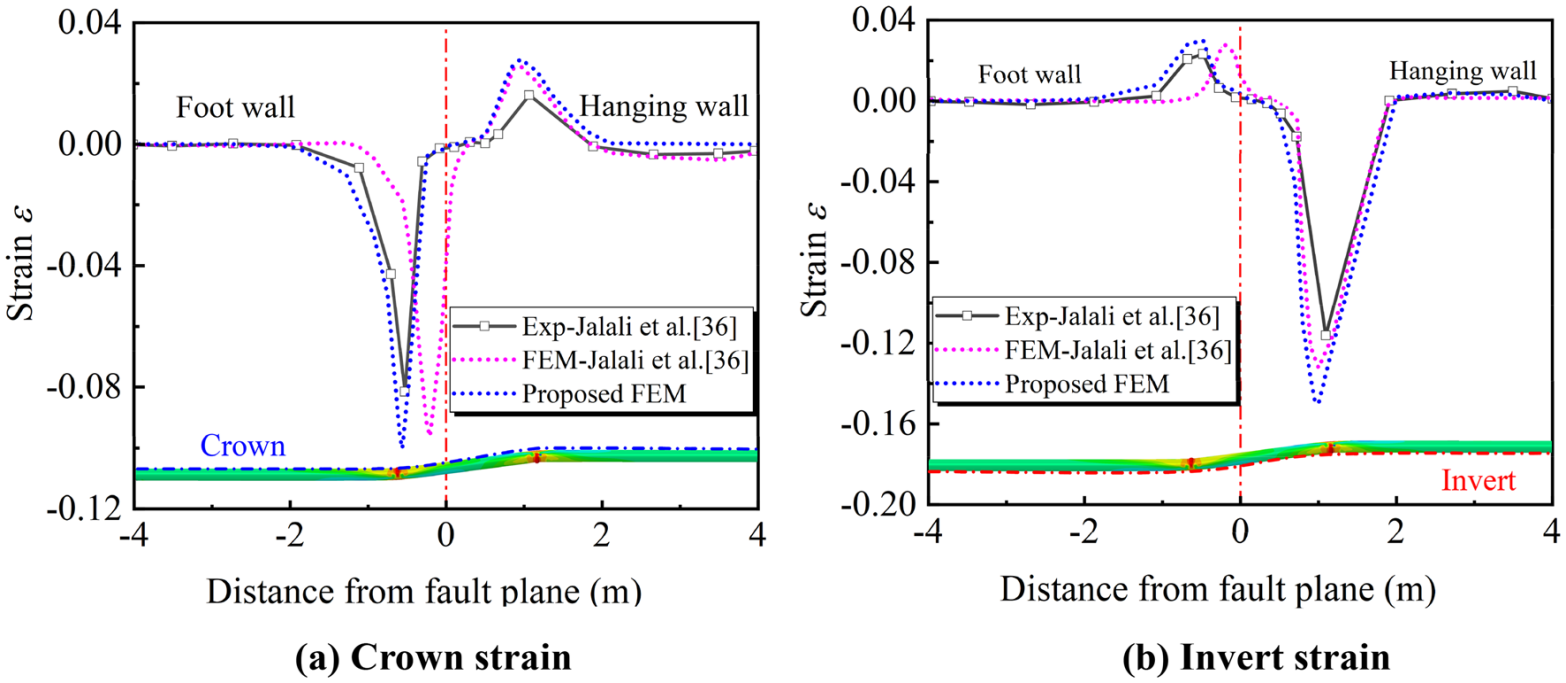
Strain distribution along the pipeline.

Taking the parameters in Table 2 into the proposed FE model for calculation and the result is shown in Fig. 7. The strains of the proposed FE model at the pipe crown and invert and the experimental measurements obtained by Jalali et al.^35^ are compared. It can be seen that the locations of the peak strain agree well with the experiment. Both of them have similar strain distribution rules, which produce large local tensile and compressive strains within 1 m on both sides of the fault, resulting in buckling or cracking of the pipe wall. The errors of strain values and potential failure positions are less than 10%. In summary, the FE model proposed in this paper can well analyze the local buckling behavior of the buried pipeline under oblique-reverse fault displacement.

## Results and discussion

### Potential location of local buckling

Pipe-fault crossing geometry, crossing angle *β* and fault dip angle *ψ*, is the primary parameter affecting the pipeline mechanical behavior. *β* can be modified during the pipeline design within the route selection procedure. According to Refs^6,36,37^, When *β* = 90°, the axial strain of the pipeline under the fault displacement is the largest, and the pipeline is most prone to failure. Thus, *β* = 90° was taken. Meanwhile, *ψ* = 60° was taken as the other parameter of the pipe-fault crossing geometry, which is more dangerous for the pipeline^2^.

**Figure 8:**
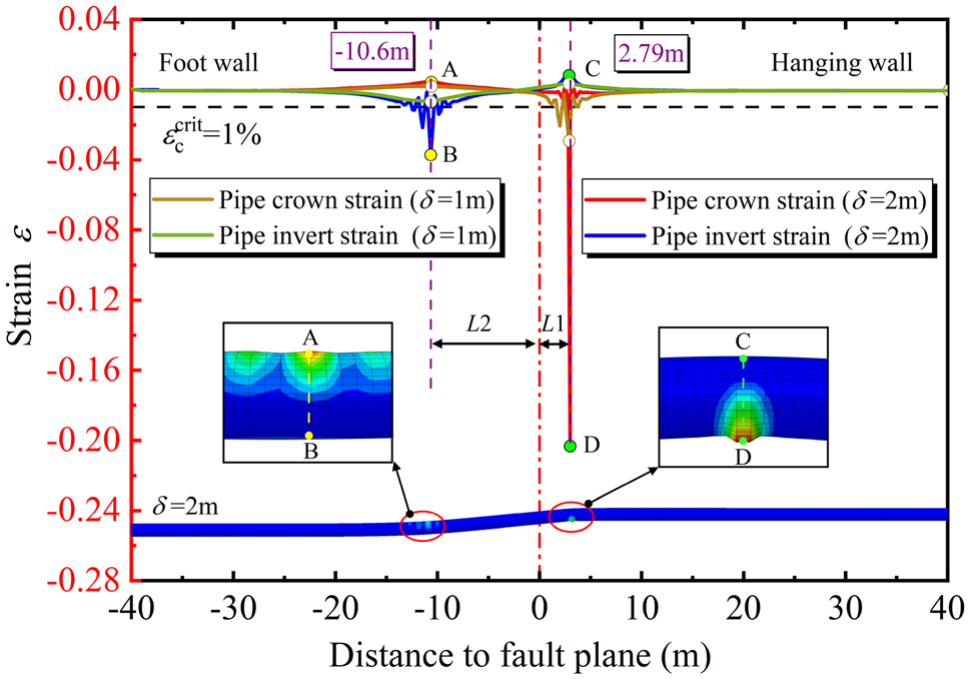
Pipe strain distributions along the pipeline under oblique-reverse displacement (*β* = 90°, *ψ* = 60°, *h* = 1.2 m, *t* = 22 mm, *D* = 1.219 m, *P* = 12 MPa).

To determine the length local buckling position, the pipe deformation and the axial strain distributions along the pipeline under the oblique-reverse displacement are shown in Fig. 8. We can see two locations of the strain concentration on the pipeline (cross-section A-B and cross-section C-D) located in the hanging and foot wall, respectively. With $\delta$ increases from $\delta$ = 1 m to $\delta$ = 2 m (there is no obvious strain concentration when $\delta$ = 0.5 m, for brevity, the pipe strain when $\delta$ = 0.5 m is not given in Fig. 8), locations of the strain concentration remain unchanged, and the pipe strain along the pipeline increases gradually, especially on cross-sections A-B and C-D. For the X80 pipeline, compared with the tensile strain, the compressive strain dominates the pipeline failure under the oblique-reverse fault displacement^2^. The compressive strain on the cross-section A-B and cross-section C-D easily exceeds *ε*_c_^crit^ under the oblique-reverse fault displacement, so the cross-sections A-B and C-D are the potential local buckling locations along the pipeline.

It should be noted that the distance from the potential buckling location on the hanging and foot wall to the fault plane (*L*1, *L*2) is different because the soil resistance on the hanging wall is larger than that on the foot wall. The distance from cross-section C-D to the fault plane is *L*1 = 2.79 m, while cross-section C-D is *L*2 = 10.6 m. The location of the cross-section C-D is closer to the fault plane. Additionally, the pipeline compressive strain on the cross-section C-D is larger and firstly exceeds the *ε*_c_^crit^.

When the pipeline is subjected to the reverse or strike-slip fault displacement, the vertical or lateral movement of the hanging wall makes the circumferential local buckling position located at the 0°–90° trace and 90°–270° trace of the pipeline, respectively. However, under the oblique-reverse fault displacement, the circumferential local buckling position of the pipeline is no longer located at the 0°–90° trace and 90°–270° trace anymore, due to the 3-D movement of the hanging wall.

**Figure 9:**
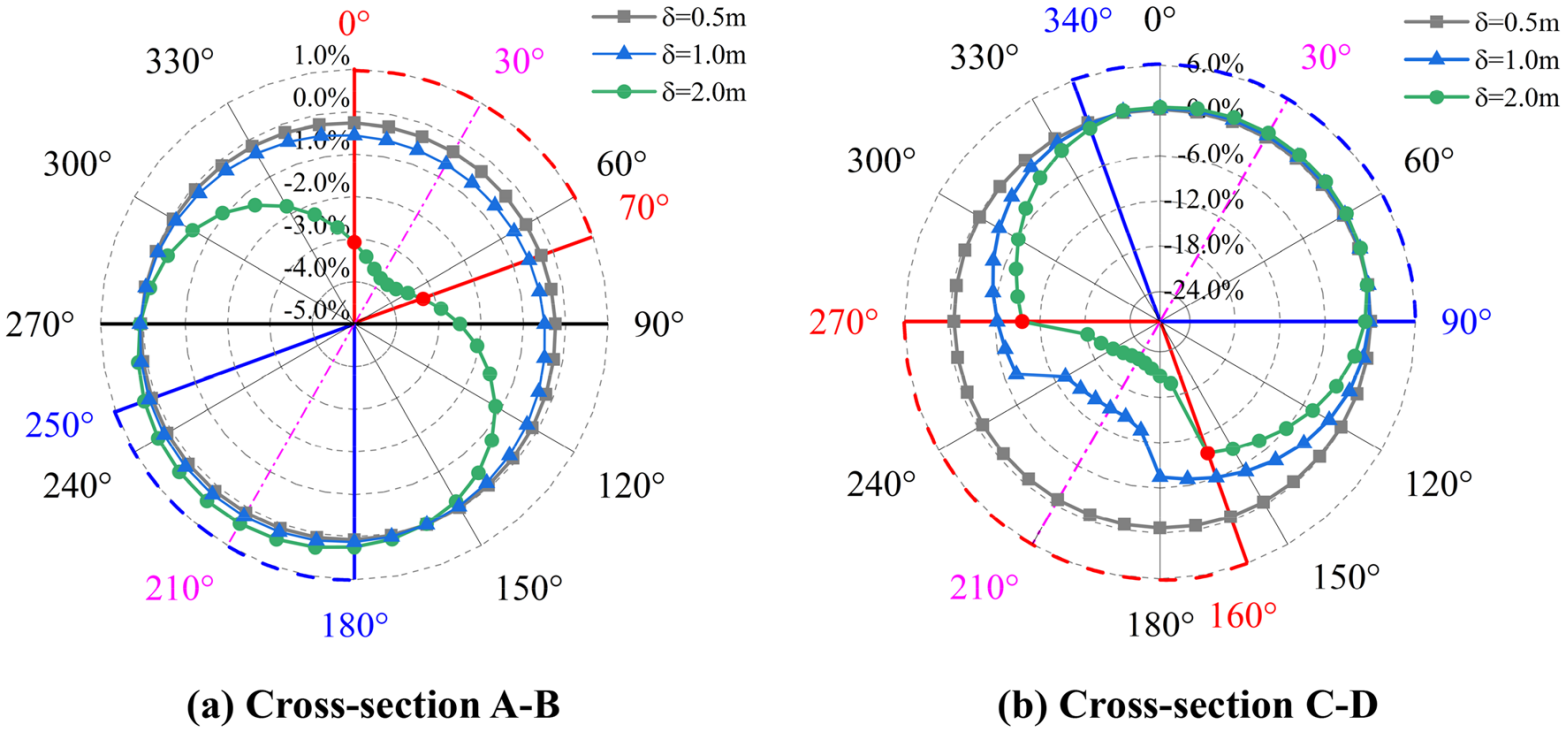
Variation of the Cross-section strain under oblique-reverse displacement (*β* = 90°, *ψ* = 60°, *h* = 1.2 m, *t* = 22 mm, *D* = 1.219 m, *P* = 12 MPa).

To determine the circumferential local buckling position, the evolution of cross-section strains under oblique-reverse fault is described in Fig. 9 by analyzing the cross-sections A-B and C-D of the pipeline. We note that with the increase of $\delta$, compressive and tensile strains of the pipeline cross-sections A-B and C-D increase gradually. At the cross-section A-B, when $\delta$ increases from $\delta$ = 0.5 m to $\delta$ = 2.0 m, *ε*_c_ in the 0°–70° area of the pipeline sharply increases. The range from 0° to 70° is the compressive zone (local buckling zone), while 180°–250° is the tensile zone accordingly. The peak compressive and tensile positions of the pipeline are about 30° and 210°, respectively.

Moreover, at the cross-section C-D, the compressive and tensile strains are more sensitive to $\delta$. With the increase of $\delta$, compressive and tensile zones expand. The compressive zone is in the range from 160° to 270°, and the tensile zone is in the range from 340° to 90°. Although the range of compressive and tensile zones expends, the peak compressive and tensile locations stabilized at 210° and 30°, respectively. Consequently, the 30°–210° trace is the circumferential local buckling position under the 3-D oblique-reverse fault displacement.

### Local buckling developing

#### Pipe strain under different fault types

**Figure 10:**
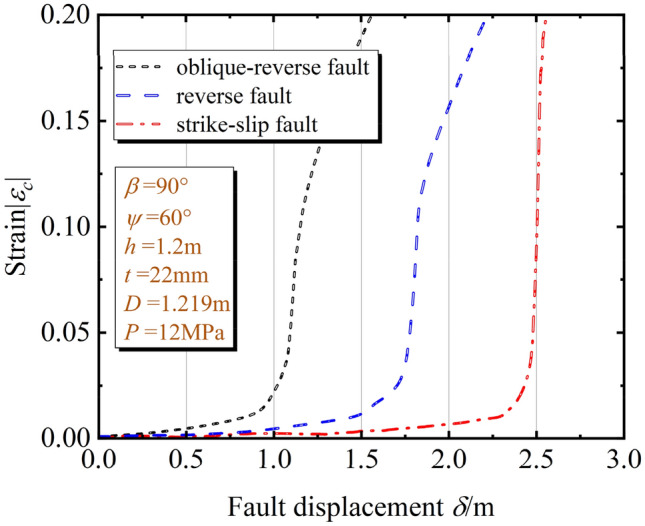
Pipeline compressive strain on the maximum strain position under different fault types.

To determine the developing process of the pipe local buckling, the pipe compressive strain on the maximum strain position and fault displacement relationship is studied. When the pipeline is subjected to the oblique-reverse fault, the stress on the pipeline is more complex as the hanging wall moves in X, Y, Z directions. Under the same operating condition (*β* = 90°, *ψ* = 60°, *h* = 1.2 m, *t* = 22 mm, *D* = 1.219 m, *P* = 12 MPa), the pipe compressive strain is more sensitive to the oblique-reverse fault displacement compared with single faults (strike-slip fault, reverse fault), as shown in Fig. 10. Under the same fault displacement, the pipe strain is larger and the pipe local buckling is more serious under the oblique-reverse fault displacement.

The local buckling developing process under the oblique-reverse fault is significantly accelerated. Using the results of pipe local buckling under single fault to evaluate the pipeline local buckling under composed fault is not safe enough. Consequently, the developing process of the pipe local buckling should be investigated when the pipeline is subjected to the oblique-reverse fault displacement.

#### Pipe local buckling developing process

Based on “Potential location of local buckling” section, the pipeline compressive strain on the cross-section C-D at 210° is more sensitive to the fault displacement and firstly exceeds the *ε*_c_^crit^, which is the riskiest location. Therefore, the local buckling developing process is researched by analyzing the compressive strain on cross-section C-D at 210°.

**Figure 11:**
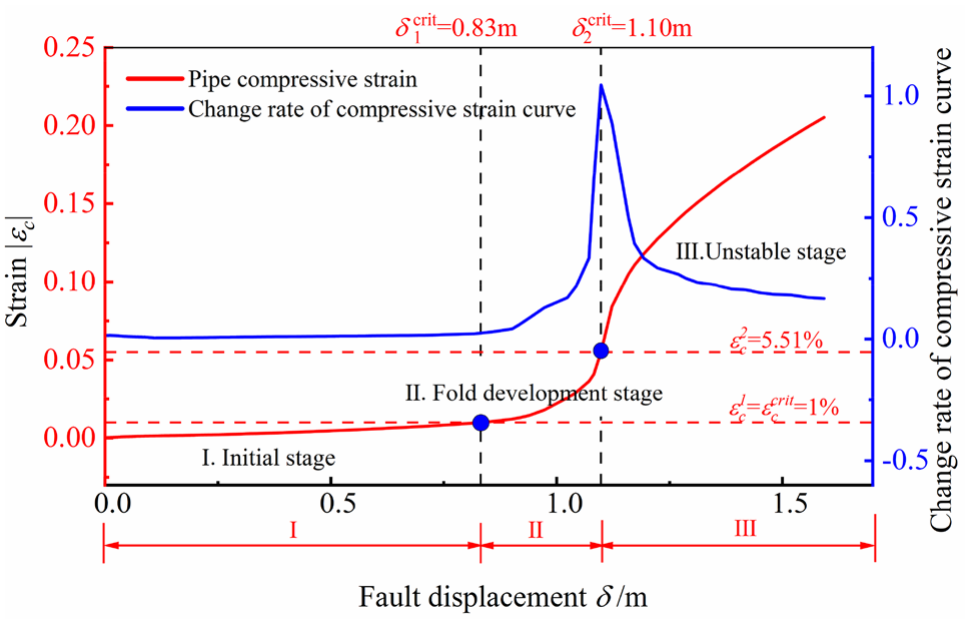
Evolution of the compressive strain under the oblique-reverse fault displacement (*β* = 90°, *ψ* = 60°, *h* = 1.2 m, *t* = 22 mm, *D* = 1.219 m, *P* = 12 MPa).

**Figure 12:**
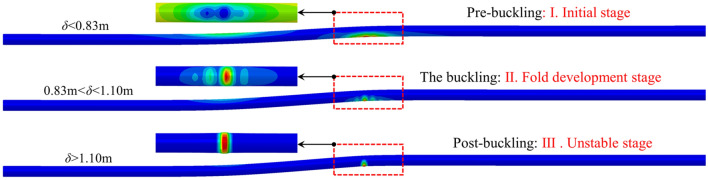
Pipeline shapes of different local buckling stages.

Figure 11 describes the variation of the compressive strain on cross-section C-D at 210° under the oblique-reverse fault displacement. According to the change rate of compressive strain, there are three stages of the developing process of pipe local buckling obviously, which are defined as three stages: pre-buckling:I. initial stage, the buckling: II. fold development stage, post-buckling: III. unstable stage. The developing process of pipe local buckling is consistent with the local buckling mechanism of the cylindrical shell, showing an obvious stage feature. Figure 12 shows shapes of the pipeline in three stages, showing the developing process of pipeline local buckling under the 3-D oblique-reverse fault displacement.

To quantitatively describe three stages of the local buckling, critical fault displacements *δ*^crit^ and critical strains of different stages are determined based on the change rate of the compressive strain. The *δ*^crit^ is defined as the fault displacement transformed between different stages, which is an objectively existing value during the local buckling developing process based on the theory in “Pipe local buckling mechanism” section. In the oblique-reverse fault operating condition, the critical fault displacement from stage I to II is *δ*_1_^crit^ = 0.83 m, and the critical fault displacement from stage II to III is *δ*_2_^crit^ = 1.10 m based on the change rate of the compressive strain, which is smaller than *δ*_1_^crit^, *δ*_2_^crit^ obtained in the single fault operating condition. The local buckling developing is more sensitive to the oblique-reverse fault displacement.

When $\delta$ < *δ*_1_^crit^ = 0.83 m, the local buckling is in the initial stage, corresponding to the linear elastic stage in Fig. 2. As $\delta$ increases, the compressive strain increases linearly and there is no obvious deformation of the pipeline in the potential local buckling location until $\delta$ exceeds *δ*_1_^crit^. When the local buckling experiences the initial stage, the pipeline can operate safely without manual intervention.

When 0.83 m = *δ*_1_^crit^ < $\delta$ < *δ*_2_^crit^ = 1.10 m, the local buckling develops to the fold development stage, and the folds gradually develop in the potential local buckling location, corresponding to the load dropped stage in Fig. 2. The CSA-Z662-2019^27^ considers that the local buckling failure of the pipeline has occurred. Although the local buckling zone has a certain plastic deformation, the local buckling zone remains stable and can still bear a certain fault displacement. The local buckling zone reaches the stable limit state when $\delta$ = *δ*_2_^crit^ = 1.10 m. When the local buckling experiences the fold development stage, it is necessary to conduct real-time monitoring at the local buckling position and take protective measures to prevent the development of local buckling from entering the unstable stage.

When $\delta$ > *δ*_2_^crit^ = 1.10 m, with $\delta$ increases, the local buckling zone of the pipeline loses its structural stability and buckles seriously, and the local buckling develops to the unstable stage, corresponding to the flow deformation stage in Fig. 2. The pipeline compressive strain increases rapidly even under small $\delta$, and the local buckling develops to an unstable state, which leads to pipeline leakage, resulting in big economic and environmental losses. When the local buckling experiences the unstable stage, the local buckling segment should be replaced or take other measures to prevent pipeline leakage.

Based on the analysis above, the local buckling developing process of the pipeline under oblique-reverse fault displacement can be defined as:

$$\left\{ {\begin{array}{*{20}c} {{\text{I}}.} & {{\text{Initial}}\;{\text{stage}}} & {\delta \le \delta_{1}^{{\text{ crit}}} } \\ {{\text{II}}.} & {{\text{Fold}}\;{\text{development}}\;{\text{stage}}} & {\delta_{1}^{{\text{ crit}}} < \delta \le \delta_{2}^{{\text{ crit}}} } \\ {{\text{III}}.} & {{\text{unstable}}\;{\text{stage}}} & {\delta > \delta_{2}^{{\text{ crit}}} } \\ \end{array} } \right.$$.

### Parameter analysis of local buckling

In actual engineering, pipelines crossing different areas are usually adopted with different *P*, *D*/*t*, and *h* (*D* = 1.219 m) to meet the goal of economic laying and safe operation. The different pipeline parameters may result in different effects on the local buckling of the pipeline. However, the circumferential local buckling position depends on the soil resistance under the pipeline and the fault displacement. It is less sensitive to the change of parameters of *P*, *D*/*t*, and *h*. Therefore, the influence of *P*, *D*/*t*, and *h* on the local buckling of the pipeline (length location of the potential local buckling and developing process) under the oblique-reverse fault displacement is analyzed systematically.

Moreover, the influence of *P*, *D*/*t*, and *h* on the pipeline under the reverse and strike-slip fault is also studied as a control group using the proposed FE model, and the *δ*^crit^ under reverse fault and strike-slip fault displacement is determined, respectively.

#### Influence of internal pressure

Internal pressure *P* of the pipeline is an important parameter that affects the local buckling behavior. Referring to the design operating pressure of Line D, the upper limit of pipeline internal pressure *P*_max_ = 12 MPa, and *P* = 0 MPa, 3 MPa, 6 MPa, 9 MPa, 12 MPa were taken.

**Figure 13:**
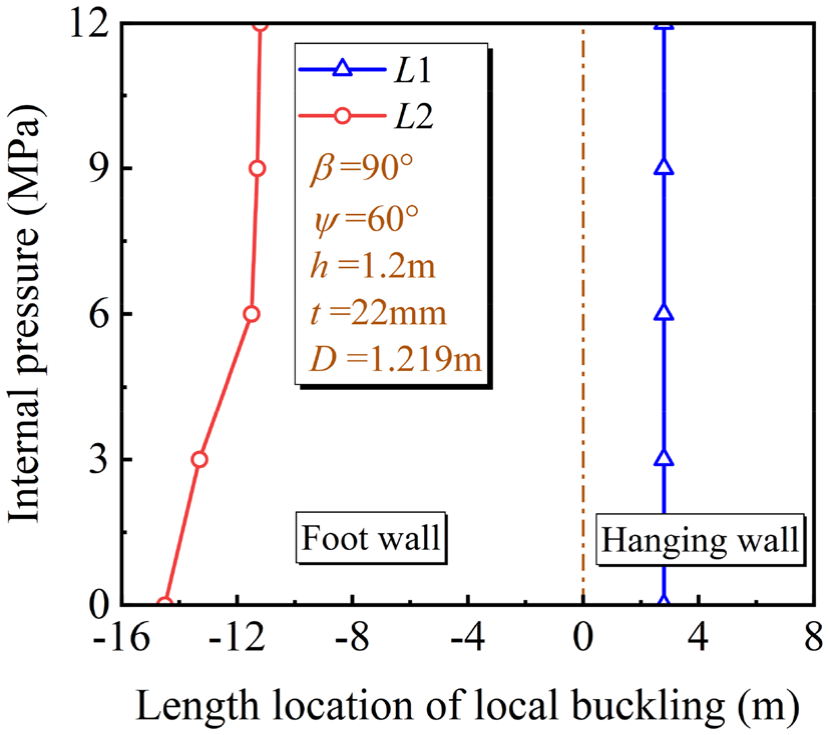
Local buckling location under different *P.*

For the length location of the local buckling, Fig. 13 shows the location of the potential local buckling under different *P*. With the increase of *P*, the deformation stiffness of the pipeline is enhanced, the potential location of local buckling in the foot wall is gradually moving to the fault plane. However, the potential local buckling location in the hanging wall is almost unchanged. During the oblique-reverse fault movement, the pipeline in the hanging wall is subjected to the soil resistance under the pipeline and the lateral, while the pipeline in the foot wall is subjected to the soil resistance above the pipeline and the lateral. In the hanging wall, compared with the pipeline stiffness, the soil resistance under the pipeline dominates the pipeline deformation. The change in the pipeline stiffness has little influence on the potential location of local buckling in the hanging wall. On the contrary, in the foot wall, compared with the soil resistance above the pipeline, the pipeline stiffness dominates the pipeline deformation and the potential local buckling position will change with the pipeline stiffness accordingly.

**Figure 14:**
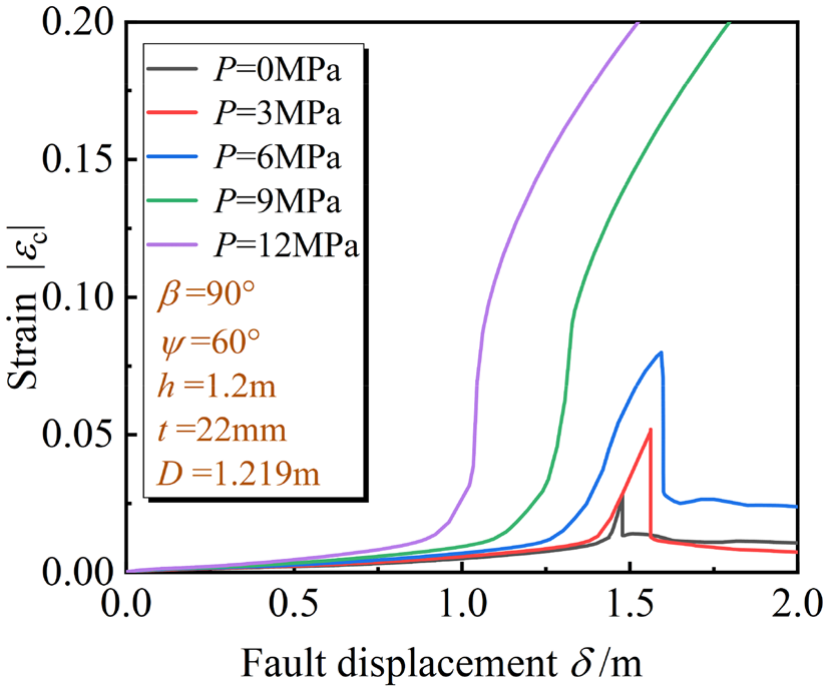
Variation of *ε*_c_ with $\delta$ under different *P.*

**Figure 15:**
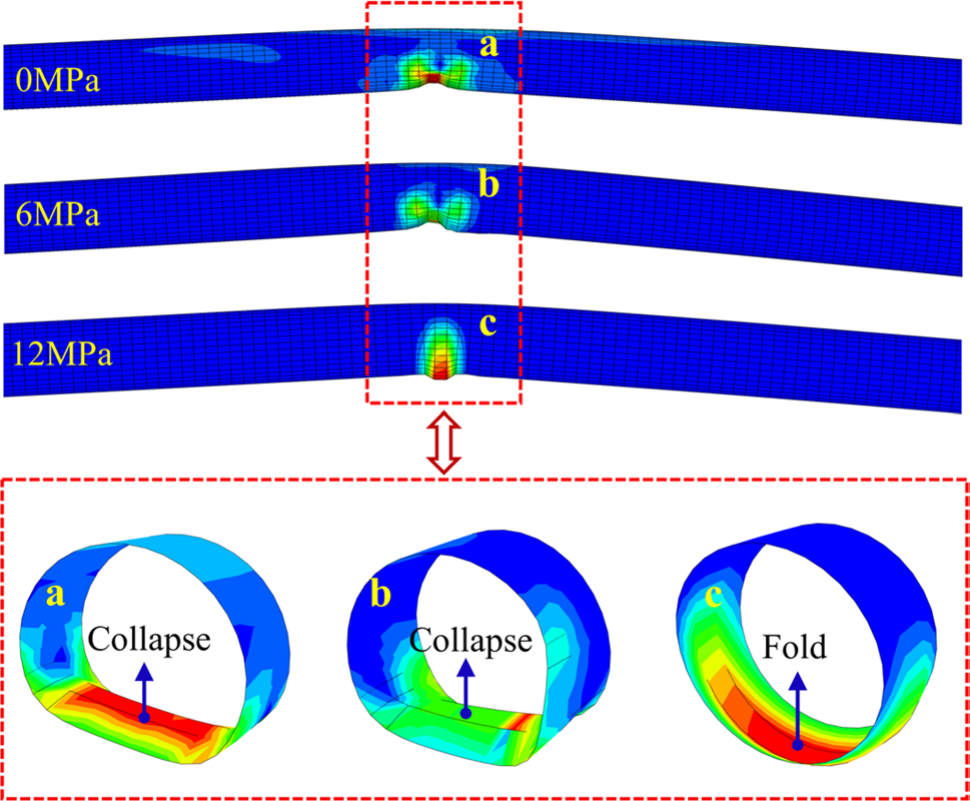
Local buckling shapes in unstable stage under different *P.*

For the developing process of the local buckling, Fig. 14 shows *ε*_c_ of the pipeline in the hanging wall with $\delta$ under different *P*. It can be seen that when *P* > 6 MPa, *ε*_c_ increases gradually with $\delta$ increases; when *P* < 6 MPa, *ε*_c_ increases with $\delta$ increases firstly, and then *ε*_c_ experiences a sharp decrease when $\delta$ is about 1.5 m. The sharp decrease of *ε*_c_ represents the phenomenon of local buckling zone collapsing to inside of the pipeline, which is shown as the inflection point in Fig. 14. Figure 15 shows the local buckling shapes of the unstable stage under different *P*. With the increase of *P*, the deformation stiffness of the pipeline is enhanced, and the form of local buckling gradually changes from collapsing to folding. (Collapsing means that the local buckling segment collapses to the pipe inside, and folding means that the local buckling segment folds to the pipe outside).

The *δ*^crit^ under different fault types and *P* are shown in Table 3. The *δ*^crit^ obtained from the oblique-reverse fault is smaller than that obtained from the single fault even under different *P*, the development of three stages of pipe local buckling under the oblique-reverse fault is significantly accelerated. The pipe local buckling is more sensitive to the change of $\delta$, and the pipeline is easier to enter the unstable stage when the pipeline is subjected to the oblique-reverse fault displacement, making the pipeline more dangerous.

**Table 3:** *δ*^crit^ under different fault types and *P.*

Stage	*P* (MPa)					
		0	3	6	9	12
I. Elastic stage	*δ*_1_^crit^	1.15 m	1.12 m	1.11 m	1.05 m	0.83 m
		(1.89 m)	(1.81 m)	(1.75 m)	(1.63 m)	(1.43 m)
II. Fold development stage		[2.45 m]	[2.42 m]	[2.40 m]	[2.35 m]	[2.26 m]
	*δ*_2_^crit^	1.48 m	1.56 m	1.58 m	1.31 m	1.11 m
III. Unstable stage		(2.23 m)	(2.1 m)	(2.03 m)	(1.91 m)	(1.70 m)
		[2.76 m]	[2.72 m]	[2.71 m]	[2.65 m]	[2.50 m]

*a* (*b*) [*c*]. *a*, *b*, *c* are *δ*^crit^ under the oblique-reverse, strike-slip and reverse fault displacement, respectively.

As also shown in Table 3, under the oblique-reverse fault, with *P* increasing from 0 to 12 MPa, *δ*_1_^crit^ and *δ*_2_^crit^ decrease gradually**,** decreasing about 25% and 28%, respectively. As *P* increases, the compressive strain becomes more sensitive to the $\delta$, and the local buckling enters the unstable stage easier with larger *P* under the oblique-reverse fault displacement.

#### Influence of diameter thickness ratio

Diameter thickness ratio *D*/*t* directly affects the deformation stiffness of the pipeline. Thicknesses of the pipe wall *t* = 18.4 mm, 22 mm, 26.4 mm, 33 mm, 44 mm were selected (*D* = 1.219 m), *D*/*t* = 68, 55, 46, 37, 28 accordingly.

**Figure 16:**
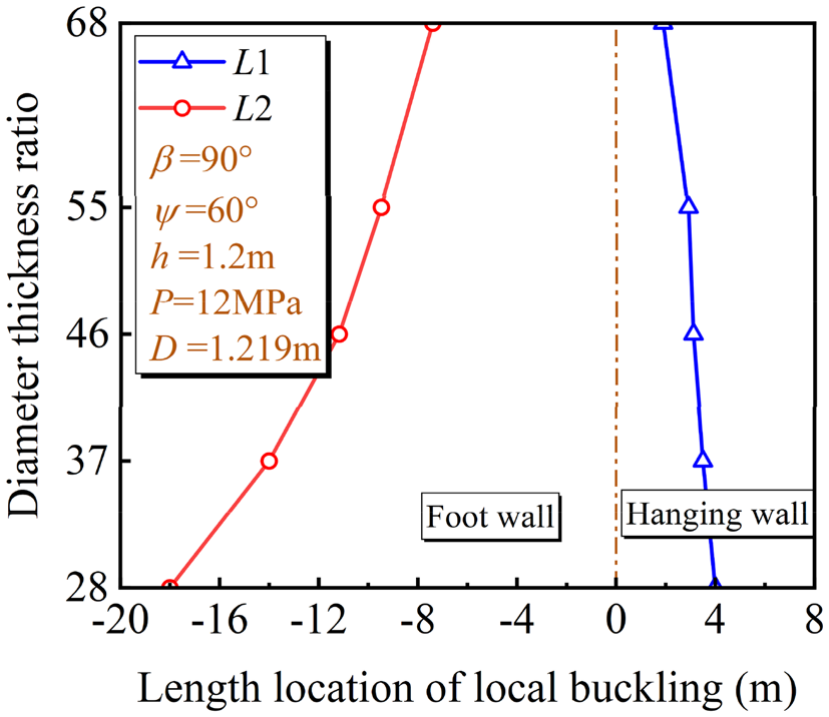
Local buckling location under different *D*/*t.*

For the length location of the local buckling, with the increase of *D*/*t*, the pipeline deformation stiffness decreases gradually, and the pipeline is more likely to deform under the fault displacement. The change rule of potential local buckling locations under different *D*/*t* is shown in Fig. 16. As *D*/*t* increases, the potential local buckling location of the pipeline in the hanging and foot wall gradually moves close to the fault plane. With the change of *D*/t, the deformation stiffness of the pipeline changes greatly. Compared with the soil resistance, the *D*/t dominates the pipeline deformation and the potential local buckling locations change with *D*/t accordingly.

**Figure 17:**
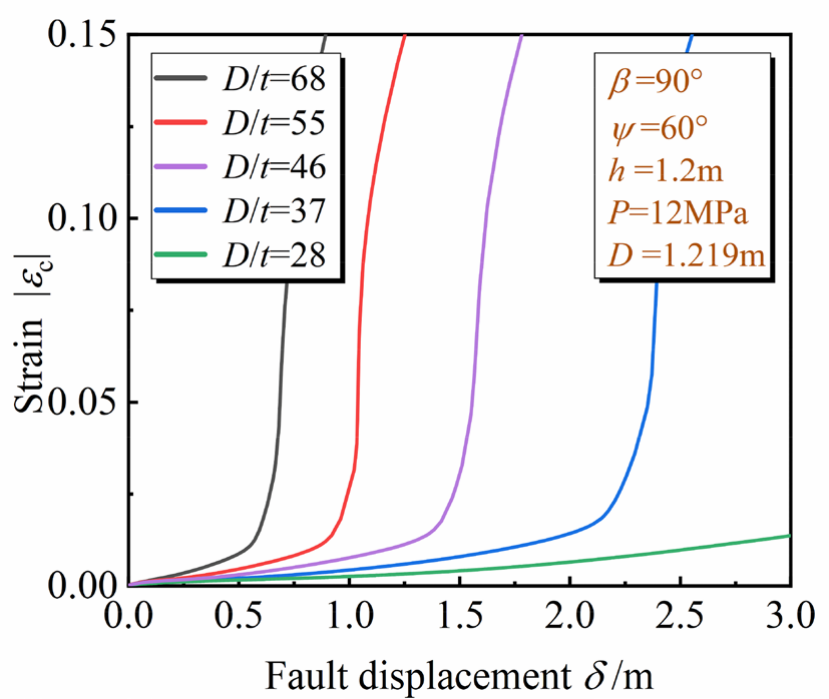
Variation of *ε*_c_ with $\delta$ under different *D*/*t.*

For the developing process of the local buckling, Fig. 17 describes *ε*_c_ of the pipeline in the hanging wall with $\delta$ under different *D*/*t*. It can be concluded that *ε*_c_ is more sensitive to $\delta$ with *D*/*t* increasing and the pipeline with smaller *D*/*t* can bear a larger oblique-reverse displacement fault without local buckling failure.

The *δ*^crit^ under different fault types and *D*/*t* are shown in Table 4. The *δ*^crit^ obtained from the oblique-reverse fault is smaller than that obtained from the single fault even under different *D*/*t*, the development of three stages of pipe local buckling under the oblique-reverse fault is also significantly accelerated. The pipe local buckling is more sensitive to the change of $\delta$, and the pipeline is easier to enter the unstable stage when the pipeline is subjected to the oblique-revere fault displacement, making the pipeline more dangerous.

**Table 4:** *δ*^crit^ under different fault types and *D*/*t.*

Stage	*D*/*t*					
		28	37	46	55	68
I. Initial stage	*δ*_1_^crit^	OCR	2 m	1.27 m	0.83 m	0.49 m
		(OCR)	(2.6 m)	(1.87 m)	(1.43 m)	(1.05 m)
II. Fold development stage		[OCR]	[OCR]	[2.62 m]	[2.26 m]	[1.92 m]
	*δ*_2_^crit^	OCR	2.40 m	1.57 m	1.11 m	0.69 m
III. Unstable stage		(OCR)	(2.9 m)	(2.13 m)	(1.70 m)	(1.32 m)
		[OCR]	[OCR]	[OCR]	[2.50 m]	[2.11 m]

*a* (*b*) [*c*]. *a*, *b*, *c* are *δ*^crit^ under the oblique-reverse, strike-slip and reverse fault displacement, respectively. OCR out of the considered range.

As also seen in Table 3, under the oblique-reverse fault, the smaller *D*/*t* is, the bigger fault displacement the pipeline can bear without local buckling failure. As *D*/*t* increases from 28 to 68, the *δ*_1_^crit^ and *δ*_2_^crit^ decrease obviously, decreasing about 75% and 71%, respectively. When *D*/*t* = 28, the local buckling is always in the initial stage, even under fault displacement corresponding to *I* = X. The pipeline characterized by small *D*/*t* can effectively slow down the development of local buckling under the oblique-reverse fault displacement.

#### Influence of burial depth

Burial depth *h* determines the pipe trench size, an important parameter in the trench design. Referring to Ref^30^, burial depths *h* = 0.9 m, 1.2 m, 1.5 m, 1.8 m, 2.1 m were selected to analyze the influence of *h* on local buckling.

**Figure 18:**
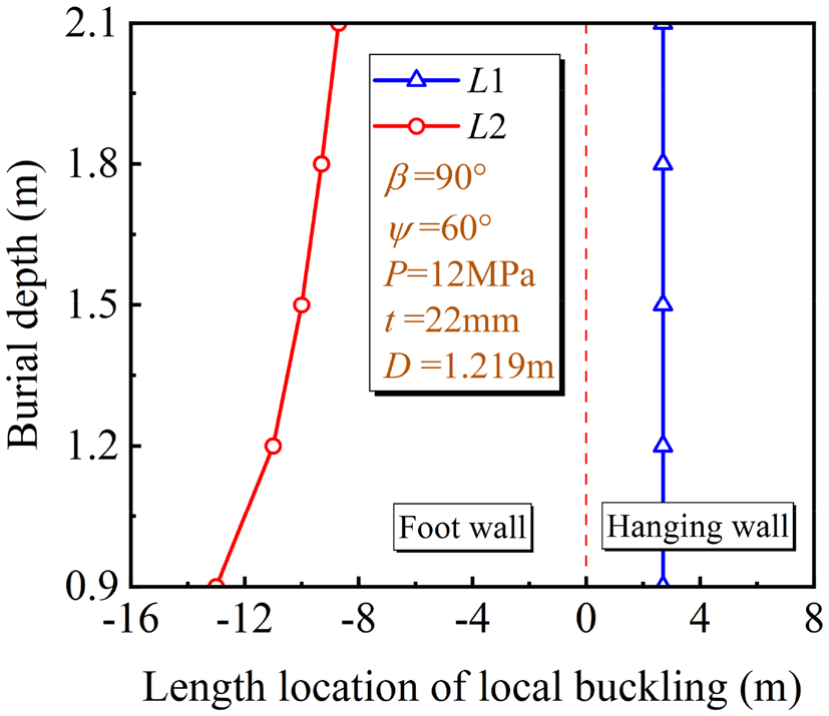
Local buckling location under different *h.*

For the length location of the local buckling, Fig. 18 describes the different potential local buckling locations under different *h*. With the increase of *h*, the potential local buckling location of the pipeline in the foot wall moves to the fault plane, while nearly no change is observed for the potential local buckling location of the pipeline in the hanging wall. As *h* increases, the soil resistance above the pipeline increases, and the potential local buckling position in the foot wall change with *h* accordingly. However, the soil resistance under the pipeline is hardly affected, and the potential local buckling position changes little.

**Figure 19:**
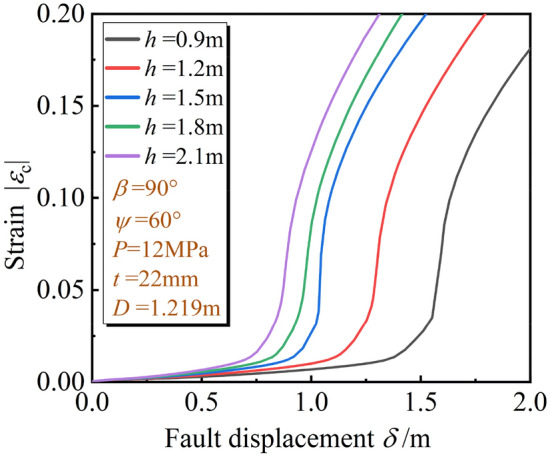
Variation of *ε*_c_ with $\delta$ under different *h.*

For the developing process of the local buckling, Fig. 19 illustrates the *ε*_c_ of the pipeline in the hanging wall. With the increase of *h*, although the potential local buckling location in the hanging wall remains unchanged, the movement resistance of the pipeline increases, and the *ε*_c_ is more sensitive to the $\delta$. The pipeline is more prone to experience local buckling.

The *δ*^crit^ under different fault types and *h* are shown in Table 5. The *δ*^crit^ obtained from the oblique-reverse fault is smaller than that obtained from the single fault even under different *h*. The development of three stages of pipe local buckling under the oblique reverse fault is also significantly accelerated. The pipe local buckling is more sensitive to the change of $\delta$ and the pipeline is easier to enter the unstable stage when the pipeline is subjected to the oblique-revere fault displacement, making the pipeline more dangerous.

**Table 5:** *δ*^crit^ under different fault types and *h.*

Stage	*h* (m)					
		0.9	1.2	1.5	1.8	2.1
I. Initial stage	*δ*_1_^crit^	1.25 m	1 m	0.83 m	0.77 m	0.63 m
		(1.81 m)	(1.62 m)	(1.43 mm)	(1.31 m)	(1.27 m)
		[2.48 m]	[2.36 m]	[2.26 mm]	[2.13 m]	[2.03 m]
II. Fold development stage						
	*δ*_2_^crit^	1.59 m	1.29 m	1.11 m	0.97 m	0.86 m
III. Unstable stage		(2.37 m)	(1.98 m)	(1.70 m)	(1.55 m)	(1.50 m)
		[2.99 m]	[2.72 m]	[2.50 m]	[2.32 m]	[2.21 m]

*a* (*b*) [*c*]. *a*, *b*, *c* are *δ*^crit^ under the oblique-reverse, strike-slip and reverse fault displacement, respectively.

As also shown in Table 5, under the influence of *h*, as the *h* increase from 0.9 to 2.1 m, *δ*_1_^crit^ and *δ*_2_^crit^ decrease gradually, decreasing about 50% and 46%, respectively. Under the same $\delta$, the greater *h* of the pipeline, the easier the local buckling reaches the unstable stage. Therefore, the shallowly burial method can delay the development of the pipe local buckling caused by $\delta$.

## Conclusion

IN this paper, the local buckling behavior of the pipeline crossing oblique-reverse fault is quantitatively studied by establishing the shell and solid element nonlinear contact coupling model. The numerical results revealed the local buckling behavior of the pipeline under the oblique-reverse fault displacement. The detailed conclusions are drawn:

1. Compared with the single fault, the developing process of the local buckling is significantly accelerated and the pipeline is more dangerous under the oblique-reverse fault displacement. The developing process of the local buckling is defined as pre-buckling: I. initial stage, the buckling: II. fold development stage and post-buckling: III. unstable stage. Moreover, the critical fault displacements *δ*_1_^crit^ and *δ*_2_^crit^ are obtained. When $\delta$ < *δ*_1_^crit^, local buckling experiences the initial stage, the pipe structure remains stable. When *δ*_1_^crit^ < $\delta$ < *δ*_2_^crit^, local buckling enters the fold development, and the compressive strain experiences a sharp increase. When $\delta$ > *δ*_2_^crit^, local buckling enters the unstable stage, and the pipeline buckles seriously. When the pipe local buckling is in different developing stages, corresponding different measures should be taken.
2. There are two potential local buckling failure locations of the buried pipeline under oblique-reverse fault displacement, located on the hanging and foot wall side, respectively. Unlike the single fault, for its circumferential location, the local buckling position of the hanging wall pipeline is about 30°, and the local buckling position of the foot wall pipeline is about 210°; for its length location, it will be affected by the operating condition. With the increase of the internal pressure, diameter thickness ratio, and burial depth, the potential local buckling in the length location of the pipeline in foot wall moves to the fault plane gradually, while that of the pipeline in hanging wall changes little.
3. The local buckling behavior (potential local buckling locations, local buckling developing process) of the pipeline with different *P*, *D*/*t*, and *h* under the oblique-reverse fault displacement could provide the reference for the pipe segment to be protected during the aseismic design and for the pipe segment to be reinforced suffered from the oblique-reverse fault displacement. The length of the pipe segment to be protected or reinforced under the oblique-reverse fault displacement could be determined based on the potential local buckling locations of the pipeline. Subsequently, the local buckling degree of the pipeline could be determined based on the local buckling stages of the pipeline under the oblique-reverse fault displacement to determine the appropriate protection or reinforcement methods.

## Data Availability

The datasets generated and analyzed during the current study are available from the corresponding author on reasonable request.
